# Neurophysiological Effects of Whole Coffee Cherry Extract in Older Adults with Subjective Cognitive Impairment: A Randomized, Double-Blind, Placebo-Controlled, Cross-Over Pilot Study

**DOI:** 10.3390/antiox10020144

**Published:** 2021-01-20

**Authors:** Jennifer L. Robinson, Julio A. Yanes, Meredith A. Reid, Jerry E. Murphy, Jessica N. Busler, Petey W. Mumford, Kaelin C. Young, Zbigniew J. Pietrzkowski, Boris V. Nemzer, John M. Hunter, Darren T. Beck

**Affiliations:** 1 Department of Psychology, Auburn University, Auburn, AL 36849, USA; jay0005@auburn.edu (J.A.Y.); jem0058@auburn.edu (J.E.M.); jzb0046@auburn.edu (J.N.B.); 2 Auburn University MRI Research Center, Auburn University, Auburn, AL 36849, USA; mareid@auburn.edu; 3 Alabama Advanced Imaging Consortium, Auburn University, Auburn, AL 36849, USA; 4 Initiative for the Center for Neuroscience, Auburn University, Auburn, AL 36849, USA; dbeck@auburn.vcom.edu; 5 Department of Electrical and Computer Engineering, Auburn University, Auburn, AL 36849, USA; 6 School of Kinesiology, Auburn University, Auburn, AL 36849, USA; pwm0009@auburn.edu (P.W.M.); kyoung@auburn.vcom.edu (K.C.Y.); 7 Edward Via College of Osteopathic Medicine, Auburn, AL 36830, USA; 8 VDF FutureCeuticals, Inc., 23 Peters Canyon Road, Irvine, CA 92606, USA; zb@futureceuticals.com; 9 VDF FutureCeuticals, Inc., 2692 N. State Route 1-17, Momence, IL 60954, USA; bnemzer@futureceuticals.com (B.V.N.); jhunter@futureceuticals.com (J.M.H.)

**Keywords:** functional magnetic resonance imaging, spectroscopy, 7T, polyphenols, nutraceuticals

## Abstract

Bioactive plant-based compounds have shown promise as protective agents across multiple domains including improvements in neurological and psychological measures. Methodological challenges have limited our understanding of the neurophysiological changes associated with polyphenol-rich supplements such as whole coffee cherry extract (WCCE). In the current study, we (1) compared 100 mg of WCCE to a placebo using an acute, randomized, double-blind, within-subject, cross-over design, and we (2) conducted a phytochemical analysis of WCCE. The primary objective of the study was to determine the neurophysiological and behavioral changes that resulted from the acute administration of WCCE. We hypothesized that WCCE would increase brain-derived neurotrophic factor (BDNF) and glutamate levels while also increasing neurofunctional measures in cognitive brain regions. Furthermore, we expected there to be increased behavioral performance associated with WCCE, as measured by reaction time and accuracy. Participants underwent four neuroimaging scans (pre- and post-WCCE and placebo) to assess neurofunctional/metabolic outcomes using functional magnetic resonance imaging and magnetic resonance spectroscopy. The results suggest that polyphenol-rich WCCE is associated with decreased reaction time and may protect against cognitive errors on tasks of working memory and response inhibition. Behavioral findings were concomitant with neurofunctional changes in structures involved in decision-making and attention. Specifically, we found increased functional connectivity between the anterior cingulate and regions involved in sensory and decision-making networks. Additionally, we observed increased BDNF and an increased glutamate/gamma-aminobutyric acid (GABA) ratio following WCCE administration. These results suggest that WCCE is associated with acute neurophysiological changes supportive of faster reaction times and increased, sustained attention.

## 1. Introduction

Recent studies have demonstrated the promising effects of bioactive phytochemicals (e.g., polyphenols) on cardiovascular and endocrine health outcomes [1,2,3,4]. As such, an increasingly intriguing line of inquiry is whether materials with high contents of these compounds may also have effects on neurophysiological and psychological measures. Preliminary evidence suggests that polyphenols may have effects in these domains, particularly in aging populations [4,5,6,7]. Whole coffee cherry extract (WCCE), is a proprietary, safe [8], powdered extract of whole coffee cherries from *Coffea arabica* with high levels of polyphenols and substantially low (<2%) levels of caffeine (for a detailed composition profile, please see Table 1 of the study by Reyes-Izquierdo et al. (2013b) [9]). WCCE has been previously associated with increased serum concentrations of both circulating and exosomal brain-derived neurotrophic factor (BDNF), in addition to increased alertness and decreased fatigue [4,9,10,11,12]. BDNF is a protein synthesized in neurons and other types of cells, and it is associated with a range of neural (e.g., plasticity) [13,14,15,16] and psychological processes [17,18,19,20]. As such, BDNF may represent an important target for identifying the pathophysiological mechanisms underlying observed behavioral or cognitive effects associated with WCCE (and possibly other polyphenol-rich materials). However, there is a need for comprehensive studies that simultaneously assess cognition and neurophysiological measures in order to better understand such mechanisms [4,5].

**Figure 1:**
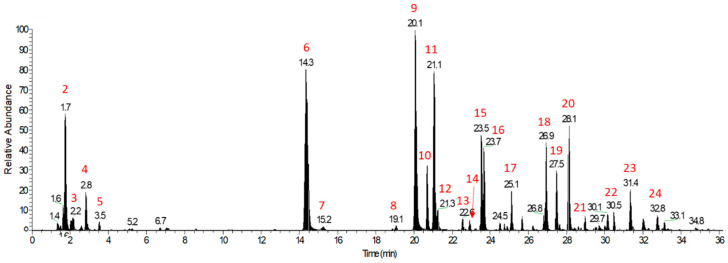
LC–MS base peak chromatogram (BPC) profile of whole coffee cherry extract (WCCE), adapted from the work of Nemzer and colleagues (under review) [27]. The major detected peaks were labelled with peak numbers ranging from 1 to 24, and the compounds corresponding to each peak are identified here: 1. gluconic acid; 2. quinic acid; 3. malic acid; 4. citric acid; 5. 2-hydroxyglutaric acid; 6. 3-O-caffeoylquinic acid (3-CQA); 7. protocatechualdehyde; 8. 3- coumaroylquinic acid (3-CoQA); 9. 5-CQA; 10. 3-feruloylquinic acid (3-FQA); 11. 4-CQA; 12. caffeic acid; 13. 5-CoQA; 14. 4-CoQA; 15. 5-FQA; 16. 4-FQA; 17. quinic acid-glucoside-R*; 18. 3-dicaffeoylquinic acid (3-DiCQA); 19. 5-DiCQA; 20. 4-DiCQA; 21. 3-Caffeoyl-5-FQA; 22. valeroylquinic acid (VQA) diglucoside-R*; 23. caffeoyl tryptophan; and 24. dimethylcaffeic acid. Other compounds identified in WCCE in the positive ion mode include pantothenic acid, trigonelline, choline, and glycerophosphocholine derivatives. Figure adapted from the work of Nemzer et al. (under review) [27]. A listing of the typical polyphenols found in WCCE can be found in Table 1.

Because of the observed beneficial effects of WCCE in the extant literature that are commensurate with evidence from other polyphenol-rich materials [7,21,22] and in light of the mounting evidence suggesting that polyphenols have neurotrophic effects [23,24,25,26], it is important to consider the chemical properties of WCCE that may drive the mechanisms involved in such effects. Recently, the phytochemical profile of WCCE was determined by a high-resolution non-targeted mass spectrometry approach (Figure 1) [27]. Importantly, one of the most abundant and widely distributed polyphenols in plants are chlorogenic acids, which are well-known for their antioxidant, anti-inflammatory, anti-hypertensive, and therapeutic properties [28,29,30]. Coffee is remarkably rich with chlorogenic acids and other polyphenols. These naturally occurring phytonutrients are concentrated during the WCCE extraction process (please see Table 1). In this study, we extended the chemical profiling of WCCE to include the antioxidant potential, as determined by five separate assays.

Interestingly, mild cognitive impairment (MCI) is associated with a reduced BDNF level [18,31]. MCI represents an intermediate stage between the expected cognitive decline of normal aging and the more serious decline of dementia. MCI is marked by problems with memory, language, thinking, and judgment that are greater than normal age-related changes. Given that earlier studies reported that WCCE may stimulate increases in BDNF [9,11], one remaining question was whether WCCE, potentially through increases in BDNF, could acutely improve cognitive function or provide protective effects in older adults who may be on the verge of or have MCI. To this end, BDNF has been associated with such changes via its effects on *N*-methyl-D-aspartate-type (NMDA) receptors [32,33,34]. Furthermore, recent evidence suggests that the long-term administration of WCCE has cognitive effects in such a population in as little as seven days and persisting for 28 days [35]. Thus, understanding the acute neurophysiological effects may provide critical mechanistic information.

Though acute, observable changes may be associated with BDNF, it is also plausible that underlying pathophysiological mechanisms may be related to some other, as-yet unidentified, neural process(es). Unfortunately, even when solely considering BDNF, methodological limitations have led to a dearth of evidence to suggest, define, support, or explain the complex and dynamic mechanistic possibilities. For example, hippocampal BDNF messenger ribonucleic acid (mRNA) expression largely depends on the neuronal excitation/inhibition balance [36], rendering it difficult to elucidate underlying mechanisms. To our knowledge, no previous study has examined neurotransmitters (e.g., glutamate and/or gamma-aminobutyric acid (GABA) concentrations) or neurometabolics concurrent with changes in BDNF levels. Therefore, the purpose of this study was to implement a comprehensive, multi-modal investigation to assess (1) the quantitative profiles of various phenolics inherent in WCCE, (2) the antioxidant properties of WCCE, and (3) the effects of WCCE in older adults on neurofunctional and neurometabolic processes while concurrently measuring associated acute cognitive and behavioral effects.

In this pilot study, representing the first of its kind, we leveraged a randomized, double-blind, placebo-controlled, within-subjects crossover design to assess the neurophysiological, neurofunctional, and cognitive effects of acute WCCE administration. Contributing to the novelty of this study, we employed advanced ultra-high field, high-resolution (i.e., submillimeter) functional magnetic resonance imaging (fMRI) and magnetic resonance spectroscopy (MRS) techniques to identify the neurofunctional and neurometabolic changes, respectively, of the acute administration of WCCE. Performing fMRI and MRS at high field strengths (e.g., 7 Tesla (7T)) offers significant advantages such as an increased signal-to-noise that allows for more sensitive assessments [37,38,39]. Likewise, using high field strengths also affords better spectral resolution, providing more robust and accurate measurements of glutamate and glutamine [40,41]. Furthermore, the utilization of a within-subjects design allows for the control of individual variability, which has recently been considered an important and necessary step toward understanding brain dynamics, favoring smaller samples with greater density of measurement [42,43,44,45,46,47,48]. We also report a complementary analysis outlining the antioxidant potential of WCCE across five separate assays. We hypothesized that WCCE would be associated with increased BDNF, improved cognitive function as measured by accuracy and reaction time during behavioral challenges, and changes in glutamate (increases) and GABA (decreases) compared to the placebo. Given the latter hypothesis, we also anticipated increased glutamate and glutamine ratios with GABA.

## 2. Materials and Methods

### 2.1. Ethics and Reproducibility

This protocol was developed at Auburn University and approved by the Auburn University Institutional Review Board (#16-391 MR 1610) in accordance with the ethical standards set forth by the Helsinki Declaration of 1975 (as revised in 1983). The study was retrospectively registered on http://clinicaltrials.gov, identifier NCT03812744 (https://clinicaltrials.gov/ct2/show/NCT03812744), and reporting guidelines for randomized, controlled trials of herbal interventions were followed where applicable [49]. For transparency purposes, we have also included figures demonstrating all data points where applicable. Future studies will be prospectively registered.

### 2.2. Participants and Recruitment

Participants were recruited from the community using fliers and through information sessions conducted in small senior groups throughout the community. Interested participants were initially screened via phone for contraindications. All potential participants were then invited to the study site for consenting and additional screening. Specifically, participants were screened for contraindications to the imaging environment, as well as memory decline using the Logical Memory (Adult Battery for Ages 16–69) section of the Wechsler Memory Scale IV (WMS) [50], the Mini-Mental State Examination (MMSE) [51,52], and the Clinical Dementia Rating (CDR) [53,54] scale. Inclusion criteria were: (1) memory complaints and memory difficulties, as verified by an informant; (2) no diagnosis of Alzheimer’s disease (and no suspected diagnosis on site by research staff); and (3) no significant cerebrovascular disease, as determined by self-reported patient history and confirmed by the informant and neurocognitive measures. Exclusion criteria were: (1) contraindications for magnetic resonance imaging (MRI) scanning, including implanted cardioverter defibrillators, any ferrous implanted metal in the body (e.g., aneurysm clips), certain types of dental work, or claustrophobia; (2) history of cardiovascular disease; and (3) those with a current or recently prescribed medication known to interfere with peripheral and/or cerebral blood flow or vascular function. Written, informed consent was obtained for all participants. Twelve participants were recruited, but 4 dropped out because of discomfort in the scanner or because of the time commitment (*n* = 8, determined by *a priori* power analyses assuming a large effect size (f = 0.60), 80% power, and a paired *t*-test design). Eight participants completed the study (4 men/4 women; 60.75 ± 2.76 (M ± SD) years of age; CDR = 0.13 ± 0.23; MMSE = 27.25 ± 0.71; and WMS = 15.88 ± 1.64), with recruitment occurring from October 2016 through 2018. Though the sample size may be considered small in the context of traditional fMRI studies, it should be noted that recent research has suggested that within-subjects designs may substantially improve power, given that larger group-based designs may not detect important differences in individual variability [42,43,44,45].

### 2.3. Procedures

**Figure 2:**
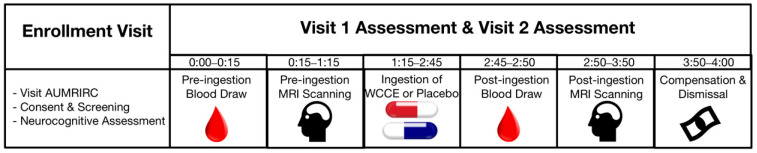
Study design overview. For the Visit 1 and Visit 2 Assessment, 0:00 indicates the participants arrival at the imaging center. Subsequent times indicate hours and minutes since arrival. AUMRIRC: Auburn University MRI Research Center.

This study was conducted as an acute, single-dose, double-blind, placebo-controlled, within-subjects crossover design (for an overview of the study design, please see Figure 2). Recruited participants were offered an opportunity to visit the Auburn University MRI Research Center (AUMRIC) prior to enrolling in the study to acclimate to the neuroimaging environment. Following initial screening and informed consent, basic demographic information was collected and neurocognitive assessments were administered (i.e., MMSE, CDR, and WMS). All participants fasted prior to the MRI study session, which occurred on a different day than the consenting and neurocognitive assessments.

#### 2.3.1. Interventions

The nutraceutical intervention used in this study was 100 mg of WCCE, commercially marketed as NeuroFactor™, manufactured by VDF FutureCeuticals, Inc. For a detailed composition profile, please see Table 1 and Figure 1. Dosage, as well as post-administration scan timing, was chosen based on previous research demonstrating significant effects on BDNF [9,11], as well as reductions in mental fatigue and higher levels of alertness [10]. Furthermore, a dosage of 100 mg of WCCE was later corroborated in a longitudinal, double-blind, placebo-controlled study in which cognitive effects were noted in as little as 7 days and persisted throughout a 28-day study period [35]. Silica oxide capsules, identical in appearance to WCCE, served as the placebo. Each capsule contained 100 mg of material. The capsules were provided by VDF FutureCeuticals, Inc., who maintained all blinding information.

#### 2.3.2. MRI Sessions

**Figure 3:**
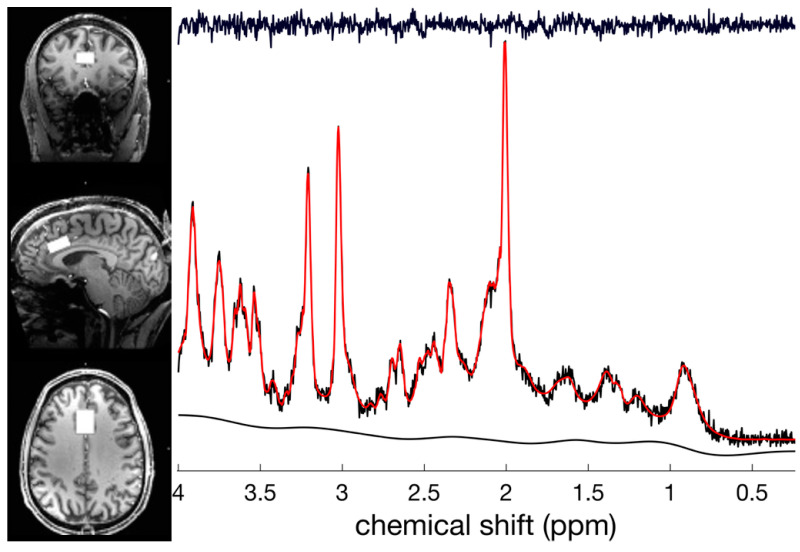
Magnetic resonance spectroscopy (MRS) voxel placement in the dorsal anterior cingulate cortex (dACC) and representative spectra. The 7 Tesla (7T) spectroscopy offers significant advantages, including more accurate assessments of glutamate and glutamine.

All MRI sessions were conducted in the morning, beginning between 8 a.m. and 9 a.m. Due to the length of the study session, participants were allowed to eat a small breakfast item between scans. This was held consistent for both sessions for all participants. Additionally, participants were asked to refrain from alcohol, caffeine, heavy exercise, and tobacco for at least 18 h prior to their scanning sessions. Blood samples were acquired upon arriving (to assess baseline BDNF levels) and 90 min following the ingestion of the study material (just prior to the post-ingestion MRI scan). Blood samples (~10 mL per draw) were collected from the antecubital veins using a butterfly catheter and captured in serum and plasma blood collection tubes containing ethylenediaminetetraacetic acid (EDTA). Samples were immediately centrifuged at 3000 rpm for approximately 15 min at 4 °C, and then they were stored at −80 °C. Participants were prepped for the scanning environment and were familiarized to the tasks that they would be performing in the scanner. Each participant had to achieve a level of mastery on the behavioral tasks outside of the scanning environment (>80% accuracy) prior to entering the MRI suite. After achieving mastery, participants underwent the scanning session. The scanning protocol consisted of the following: a localizer, gre-field mapping (36 slices; TR/TE: 400/4.92 ms; 3.1 × 3.1 × 3.0 mm voxels; 60° flip angle; and base/phase resolution: 64/100), a whole brain functional scan for registration purposes (60 slices; TR/TE: 6000/28 ms; 0.9 × 0.9 × 1.5 mm voxels; 70° flip angle; base/phase resolution 234/100; and GRAPPA acceleration factor of 3), a MPRAGE 3D high resolution structural scan (256 slices; TR/TE: 2200/2.89 ms; 0.7 mm^3^ isotropic voxels; 7° flip angle; and base/phase resolution: 256/100), MRS focused on the dorsal anterior cingulate cortex (dACC; Figure 3) (STEAM; TR/TE/TM: 10,000/5/45 ms; 25 × 20 × 12 mm voxel size; 32 averages with water suppression; 2 averages without water suppression; 4 kHz spectral bandwidth; and 2048 points), a resting state fMRI scan (37 slices; TR/TE: 3000/28 ms; 70° flip angle; base/phase resolution: 234/100; GRAPPA acceleration factor of 3; interleaved sequence; 100 volumes; and total acquisition time 5:00), and fMRI n-back and go/no-go tasks (same sequence parameters as the resting state fMRI, but the number of volumes were 151 and 142, respectively). Participants completed all scanning tasks and were escorted out of the scanning environment to the lounge area, where they immediately ingested either WCCE or the placebo. Following a designated 90-min wait time, another blood draw was conducted, and the scanning procedure was repeated. Participants were then dismissed and returned after 72 h to repeat the entire protocol with the alternate test substance. We chose a 72-h interval based on our assessment of the literature that suggested that polyphenols should be fully metabolized within this timeframe [55,56,57]. Order was randomly assigned (via a random number generator by author D.T.B.), with six individuals assigned to WCCE followed by the placebo and two assigned to the placebo followed by WCCE. Participants and investigators were blind to test substance assignment, and the sponsor was blind to participant assignment until the end of the study. Participants were compensated for their time ($75 for completing the first session and $125 for completing the second session).

#### 2.3.3. Exosomal BDNF

Exosomal BDNF was measured using a commercially available ELISA according to manufacturer’s instructions (Sigma Aldrich, St. Louis, MO, USA). Exosomes were isolated from serum aliquots and separated by precipitation using an ExoQuick™ Exosome Precipitation Kit (System Biosciences, Mountain View, CA, USA). Briefly, the supernatant was collected in a clean tube while the pellet was centrifuged for 5 min at 1500× *g* and any remaining supernatant was removed. The pellet was resuspended in 250 μL of an exosome-binding buffer (System Biosciences, Mountain View, CA, USA) and incubated in an ice bath for 5 min to complete the lytic process. BDNF contained in the exosomal lysate was measured using the previously described BDNF ELISA (Sigma Aldrich, St. Louis, MO, USA). The intra- and inter-assay coefficients of variance were 3.2% and 5.0%, respectively.

#### 2.3.4. Behavioral Tasks

Go/no-go task: The go/no-go task is designed to assess inhibitory responses. Stimuli were presented in a continuous stream, and participants were asked to perform a binary decision on each stimulus (e.g., is the stimulus in an alternating pattern with the previous stimulus?). Participants pressed a button if the stimulus met the criteria (go trial) or withheld their response if it did not (no-go trial/lures). The task was designed to have more ‘go’ than ‘no-go’ trials, enticing the participant into a pattern of responses, thus making the ‘no-go’ trials more difficult in populations with poor impulse control [58,59,60,61,62]. Accuracy and reaction time, two of our primary outcome measures, were recorded by the E-Prime (https://pstnet.com/products/e-prime/) stimulus presentation software. Stimuli were presented once every 1000 ms with an additional 500 ms intertrial interval to allow for responses. Following an instructional slide and a 4-s countdown slide, the task was presented in a block design such that 60 trials were presented followed by a 15 s rest period, which repeated a total of 3 times. A final block of 71 trials was also administered (total time = 7:05, total trials = 251 (247 requiring a response, since the first trial after any rest period did not require a response), total lures = 25, or ~10%). This task reliably activates regions of the prefrontal cortex (PFC), including the middle frontal gyrus, the inferior frontal gyrus, and the anterior cingulate [63,64]. These regions are implicated in cognitive control and response inhibition, making this an ideal task for examining the effects of WCCE on cognitive function.

N-back: The n-back task is widely used in working memory literature. It requires participants to determine if the current stimulus repeats relative to the item that occurred ‘n’ times before its onset. As such, a 1-back task would require participants to determine if the current stimulus is the same as the one previous to it, whereas a 2-back task would require participants to determine if the present stimulus is the same as 2 stimuli ago. The task becomes increasingly harder with higher numbers of stimuli that need to be recalled. We used the 1-back task as a cognitive challenge. Stimuli were presented for 1500 ms with a 500 ms intertrial interval in blocks of 50 trials for a total of 200 trials. Each block was separated by a 15 s rest period (total time = 7:25). The neural network actively involved in the n-back includes an extensive frontoparietal network, encompassing regions involved in attention and decision making [65,66]. For both cognitive tasks, accuracy, defined as the number of correct responses, and reaction time, measured in ms, were recorded by the E-Prime (https://pstnet.com/products/e-prime/) stimulus presentation software.

### 2.4. Chemical Analysis

The commercially available WCCE, marketed under the trade name of “Neurofactor™,” was standardized to 40% minimum chlorogenic acids. Neurofactor™ was manufactured and supplied by VDF FutureCeuticals, Inc. Chemical analyses were conducted by VDF FutureCeuticals, Inc. (B.V.N.). Below, we report methods for high HPLC analyses.

#### 2.4.1. Chlorogenic Acid

Samples were extracted in 50% methanol, separated by reversed-phase HPLC and detected at 325 nm. Chlorogenic acid and related compounds present in the sample were quantified using a commercially available standard as the external standard. Response factors were used to quantify the related chlorogenic acid compounds against chlorogenic acid.

#### 2.4.2. Trigonelline by HPLC

Trigonelline is naturally found in coffee plants. Trigonelline was extracted from the samples using an ammonium formate buffer, which was separated by reversed-phase HPLC with detection at 265 nm by photodiode-array detection (PDA/DAD), and quantification was calculated using a commercially available standard.

#### 2.4.3. Caffeine

Caffeine was extracted using 70% methanol, separated by reversed-phase HPLC, measured at 272 nm using PDA/DAD, and quantified using a commercially available standard. The caffeine and trigonelline contents were characterized by HPLC Agilent 1100 (Agilent Technologies) equipped with a diode array detector and quantified by UV absorbance.

#### 2.4.4. Sample Preparation for Antioxidant Measurements

Approximately 20 mg of each sample were weighed and extracted with 20 mL of ethanol/water (70:30 *v*/*v*) for 1 h at room temperature on an orbital shaker. The extracts were centrifuged at 5900 rpm, and the supernatant was used for the total antioxidant capacity assay. The total antioxidant capacity was determined by calculating the sum of the individual result against five free radicals, namely peroxyl radicals, hydroxyl radicals, peroxynitrite, superoxide anions, and singlet oxygen. All results were expressed as µM Trolox equivalent per gram (µM TE/g). Ethanol (HPLC grade) and 6-hydroxy-2,5,7,8 tetramethyl-2-carboxylic acid (Trolox) were obtained from Sigma-Aldrich (St. Louis, MO, USA).

##### Peroxyl Radicals Scavenging Capacity (ORAC Assay)

The oxygen radical absorbance capacity (ORAC) assay was conducted on the basis of a report by Huang, Ou, and colleagues [67,68], modified for the FL600 microplate fluorescence reader (Bio-Tek Instruments, Inc., Winooski, VT, USA). The FL600 microplate fluorescence reader was used with fluorescence filters for an excitation wavelength of 485 ± 20 nm and an emission wavelength of 530 ± 25 nm. The plate reader was controlled by the KC4 3.0 software, and 2,2′-Azobis (2-amidinopropane) dihydrochloride (AAPH) was used as the source for the peroxyl radical, which was generated as a result of the spontaneous decomposition of AAPH at 37 °C. AAPH was obtained from Wako Chemicals USA, Inc. (Richmond, VA, USA). Fluorescein was the chosen target protein, with a loss of fluorescence an indicator of the extent of damage from its reaction with the peroxyl radical. The protective effect of the antioxidants was measured by comparing the fluorescence time/intensity area under the curve of the sample compared with a control assay with no antioxidant compounds present. Trolox, a water-soluble analogue of vitamin E, was used as the calibration standard. Fluorescence readings were taken every minute for up to 35 min following the addition of AAPH.

##### Hydroxyl Radical Scavenging Capacity (HORAC Assay)

The HORAC assay was based on a report by Ou and colleagues (2002) [67] and modified for the FL600. Fluorescein (FL) was used as the probe. The fluorescence decay curve of FL was monitored in the absence or presence of antioxidants. The area under the fluorescence decay curve (AUC) was then integrated, and the net AUC was calculated by subtracting the AUC of the blank from that of the sample antioxidant.

##### Peroxynitrite Scavenging Capacity (NORAC Assay)

Peroxynitrite (ONOO-) scavenging was measured by monitoring the oxidation of dihydrorhodamine-123 (DHR-123) according to a modification of the method of Chung and colleagues (2001) [69]. Briefly, a stock solution of DHR-123 (5 mM) in dimethylformamide was purged with nitrogen and stored at −80 °C. A working solution with DHR-123 (final concentration (fc) of 5 µM) diluted from the stock solution was placed on ice in the dark immediately prior to study. The buffer of 90 mM sodium chloride, 50 mM sodium phosphate (pH 7.4), and 5 mM potassium chloride with 100 µM (fc) diethylenetriaminepentaacetic acid (DTPA) was purged with nitrogen and placed on ice before use. ONOO- scavenging by the oxidation of DHR-123 was measured with an FL600 microplate fluorescence reader with excitation and emission wavelengths of 485 and 530 nm, respectively, at room temperature. The background and final fluorescent intensities were measured 5 min after treatment with or without SIN-1 (fc 10 µM) or authentic ONOO- (fc 10 µM) in 0.3 N sodium hydroxide. The oxidation of DHR-123 by the decomposition of SIN-1 gradually increased, whereas authentic ONOO- rapidly oxidized DHR-123, with its final fluorescent intensity being stable over time.

##### Superoxide Anion Scavenging Assay (SORAC Assay)

The SORAC assay was based on the previously described method by Zhang and colleagues (2009) [70]. Simply, hydroethidine was used as a probe in measuring O_2_^•−^ scavenging capacity. Nonfluorescent hydroethidine was oxidized by O_2_^•−^ generated by the mixture of xanthine and xanthine oxidase to form a species of unknown structure that exhibited a strong fluorescence signal at 586 nm. The addition of SOD inhibited the hydroethidine oxidation.

##### Singlet Oxygen Scavenging Assay (SOAC Assay)

The SOAC assay was based on the previously described method by Zhang and colleagues (2009) [70]. Singlet oxygen was generated from the mixture of H_2_O_2_ and MoO_4_^2−^. Hydroethidine (HE) was used as a probe to singlet oxygen. Hydroethidine was prepared in *N,N*-dimethylacetamide (DMA) in order to make 40 μM solution. Additionally, 2.635 mM Na_2_MoO_4_ and 13.125 mM H_2_O_2_ working solutions were prepared in DMA. An HE solution (125 μL) was added to a well, followed by the addition of 25 μL of 2.635 mM Na_2_MoO_4_^2-^ and 25 μL of 13.125 mM H_2_O_2_, respectively. The plate was then transferred to an FL600 microplate fluorescence reader with excitation and emission wavelengths of 530 and 620 nm, respectively, to record the change of fluorescence intensity at 37 °C for 35 min. The addition of the samples inhibited the oxidation of hydroethidine induced by singlet oxygen.

### 2.5. Statistical Analysis Plan

#### 2.5.1. Functional Magnetic Resonance Imaging (fMRI)

FMRI data processing was carried out using FEAT (FMRI Expert Analysis Tool) Version 6.00, part of FSL (FMRIB’s Software Library, www.fmrib.ox.ac.uk/fsl) [71,72]. Prior to statistical modeling, non-brain material was removed from the data, slice timing correction was applied using Fourier-space time-series phase-shifting, motion correction was applied using MCFLIRT [73], and a high-pass temporal filter (Gaussian-weighted least-squares straight line fitting, with sigma = 50.0 s) was applied. Data from these analyses were smoothed with a Gaussian kernel of FWHM 5.0 mm. The grand-mean intensity normalization of the entire 4D dataset by single multiplicative factor was also applied. Functional images were registered to a high-resolution T1 anatomical volume and standardized to Montreal Neurological Institute (MNI) space [73,74]. Timeseries statistical analysis was carried out using FILM with local autocorrelation correction [75]. Higher-level analyses were performed with a mixed effects model where subjects were treated as random factors, and images contrasting the ‘on’ and ‘off’ conditions were generated for each task (i.e., n-back and go/no-go). For our analyses, we also used motion artifact detection (fsl_motion_outliers, a script in the FSL software suite) to create confound matrices that were subsequently used in the general linear model (GLM) analyses to completely remove the effects of severe motion volumes from the analyses without any adverse effects on the statistics. The format of the confound matrix is a separate column for each time point that is deemed to be an outlier. Within each column, the values are all zeroes except for a value of ‘1’ at the time point that is considered to be the outlier. The effect of adding this to the GLM is that it fully models all the influence of that time point with a separate parameter estimate which means that the intensities at that time point (in any voxel) have no influence on any of the other parameter estimates, effectively removing the effect of this time point from the estimation of all the effects of interest. Group *z*-statistic images were thresholded on magnitude (*z* ≥ 2.3), as well as cluster-extent determined by *z* > 2.3 and a corrected cluster significance threshold of *p* < 0.05 [76] using a within-subjects, repeated measures design (controlling for each individual’s average brain activation over their four imaging sessions to account for individual differences in global brain activity). For the go/no-go task, one participant’s pre-WCCE task data were excluded due to a scanner reconstruction error. Statistical images were generated for task, rest, and contrasts between task and rest for both go/no-go and n-back tasks. Additionally, for the go/no-go task, we examined differences between go trials and no-go trials, modelled separately.

Connectivity modeling was executed using the ‘conn’ toolbox [77] (https://www.nitrc.org/projects/conn/) for MATLAB and SPM12 [63,64,65,70,72,78] (Statistical Parametric Mapping (http://www.fil.ion.ucl.ac.uk/spm)) using standard fMRI pre-processing steps (i.e., brain extraction, slice timing correction, Gaussian smoothing (5 mm FWHM), band-pass filtering (0.008–0.09), regression of motion and physiological artifacts, registration to anatomical space, and normalization to MNI standard space). Voxel-to-voxel and seed-to-voxel connectivity maps were generated.

#### 2.5.2. Magnetic Resonance Spectroscopy (MRS)

For MRS data, spectra were analyzed using the LCModel software (version 6.3-1J) [79]. LCModel performs automatic quantification of in vivo proton MR spectra by analyzing spectra as a linear combination of model spectra from sequence-specific simulations. The water-suppressed spectra were eddy current-corrected and quantified using the unsuppressed water signal. Spectra were analyzed using the default fitting range of 0.2–4.0 ppm. One participant’s spectra had a large lipid peak, so a different fit window (1.6–4.0 ppm) was applied. Cramer–Rao lower bounds (CRLBs) were used as a measure of fit, and metabolites with CRLB > 50% were rejected from further analysis [80]. Metabolite concentrations were then calculated. All values were cerebrospinal fluid (CSF)-corrected using methods outlined in Gasparovic, Song [81]. Of note, GABA was not detected by the LCModel in two spectra (one post-WCCE and one post-placebo). Glutamate/GABA and glutamine/GABA ratios were calculated from the spectral fittings of each respective neurometabolite. We conducted paired *t*-tests in lieu of repeated measures ANOVA because of the pilot nature of this study and because the power analyses were based on main effects only, not interactions.

#### 2.5.3. Exosomal BDNF and Behavioral Data

We conducted paired *t*-tests to determine differences between timepoints (post–pre) and conditions (WCCE–placebo). Paired *t*-tests were chosen because of the pilot nature of this study and because the power analyses were based on main effects only, not interactions. Effect sizes are reported for significant results for use in planning larger studies in the future.

## 3. Results

### 3.1. FMRI: General Linear Modeling

**Figure 4:**
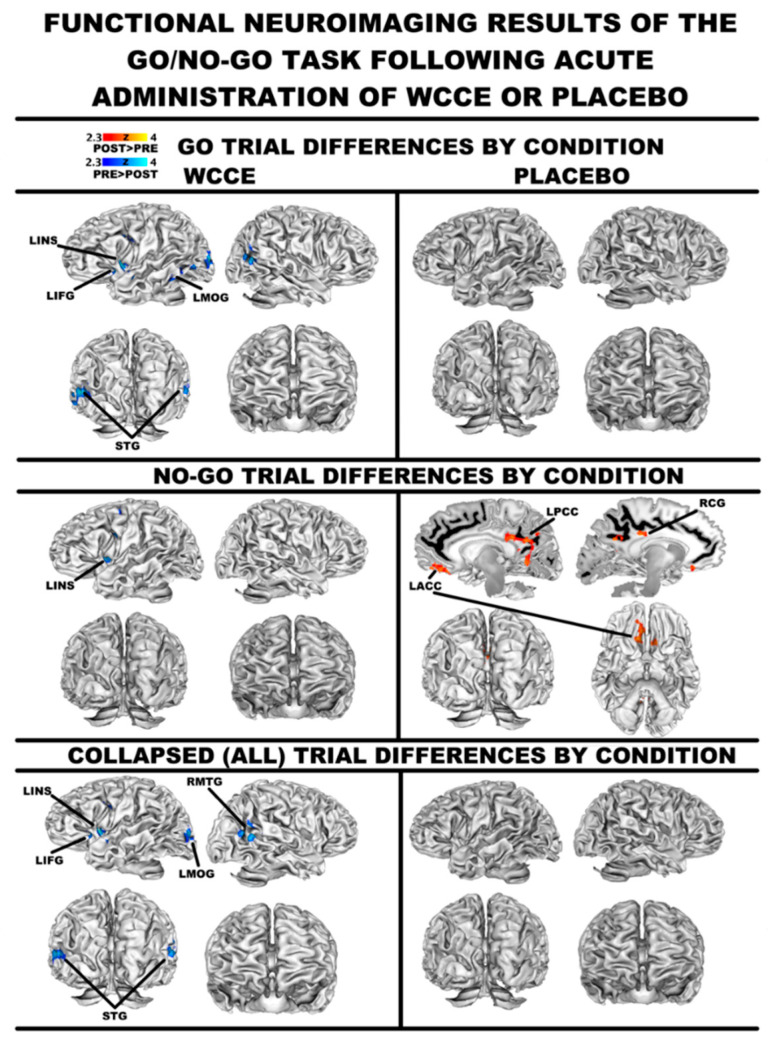
Differences in the pre–post consumption of WCCE or placebo for go trials (uppermost panel), no-go trials (middle panel), and all trials collapsed (bottom panel). In all panels, WCCE is presented on the left side, and the placebo is presented on the right. Statistic images were thresholded on magnitude (*z* ≥ 2.3), as well as cluster extent-determined by *z* > 2.3 and a corrected cluster significance threshold of *p* < 0.05. Local maxima tables for each contrast are available at https://osf.io/qypr8/. Abbreviations: LACC = left anterior cingulate cortex; LIFG = left inferior frontal gyrus; LINS = left insula; LMOG = left middle occipital gyrus; LPCC = left posterior cingulate cortex; RCG = right cingulate gyrus; and STG = superior temporal gyrus.

Go/no-go: When collapsing across trial types, WCCE ingestion was associated with a decreased blood-oxygen level dependent (BOLD) activity during go/no-go trials in the left inferior frontal gyrus (Brodmann area (BA) 9/47), the left cingulate gyrus (BA 24), bilateral superior temporal gyri (BA 22), and portions of the right middle temporal gyrus (BA 39) (Figure 3; for a listing of all coordinates associated with differences in activation patterns, please see the supplemental tables hosted at https://osf.io/qypr8/). Additionally, deactivations were noted in the left precentral and middle frontal gyri (BA 6), as well as the insula during no-go trials, while an extensive fronto-limbic network deactivated during go-trials. Post-placebo ingestion was associated with increased activity throughout key decision-making regions, namely bilateral cingulate/posterior cingulate (BA 23/24/31) during the no-go trials compared to the go trials (please see the supplemental tables hosted at https://osf.io/qypr8/). Furthermore, no-go trials elicited greater activity in bilateral anterior cingulate following the ingestion of the placebo (Figure 4).

N-back: Post-WCCE ingestion was associated with greater right posterior cingulate (BA 29/30), parahippocampal (BA 30), and culmen activity during rest periods compared to pre-ingestion (please see https://osf.io/qypr8/ for a listing of coordinates). No differences were observed for the placebo.

### 3.2. FMRI: Connectivity

**Figure 5:**
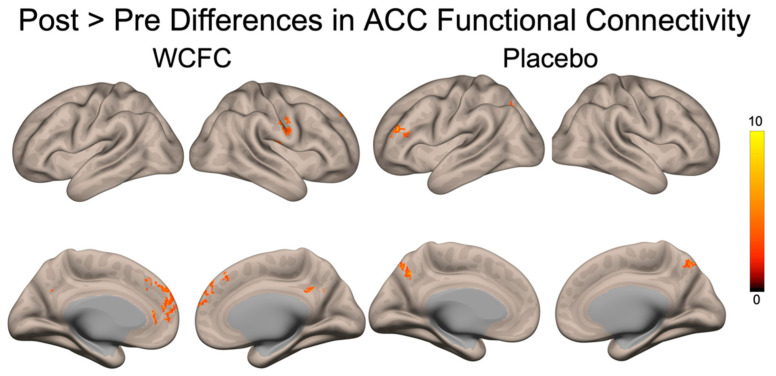
Anterior cingulate functional connectivity differences (post > pre) during the n-back task. WCCE showed greater connectivity post-consumption between the ACC and the paracingulate, the precuneus, and portions of the superior frontal gyrus and frontal poles. The placebo demonstrated a greater connectivity between the ACC and portions of the left dorsolateral prefrontal cortex, as well as the precuneus. Data were thresholded with *p*_uncorrected_ < 0.01 height threshold, *p*_FDR-corrected_ < 0.05 cluster threshold, one-tailed.

Functional connectivity differences were observed in the ACC during the go/no-go task such that following WCCE ingestion, the ACC had greater connectivity with the left superior frontal gyrus (height threshold *p*_uncorrected_ < 0.01; cluster threshold *p*_FDR-corrected_ < 0.05). Furthermore, during the n-back task, we found significant differences in post-WCCE > pre-WCCE between the ACC and the precuneus, paracingulate, bilateral superior frontal gyri, and bilateral frontal poles (one-sided *t*-test, *p*_uncorrected_ < 0.01 height threshold; *p*_FDR-corrected_ < 0.05 cluster threshold). Placebo post > pre ACC connectivity was greater with the precuneus and left inferior and middle frontal gyri (Figure 5).

### 3.3. MRS

**Figure 6:**
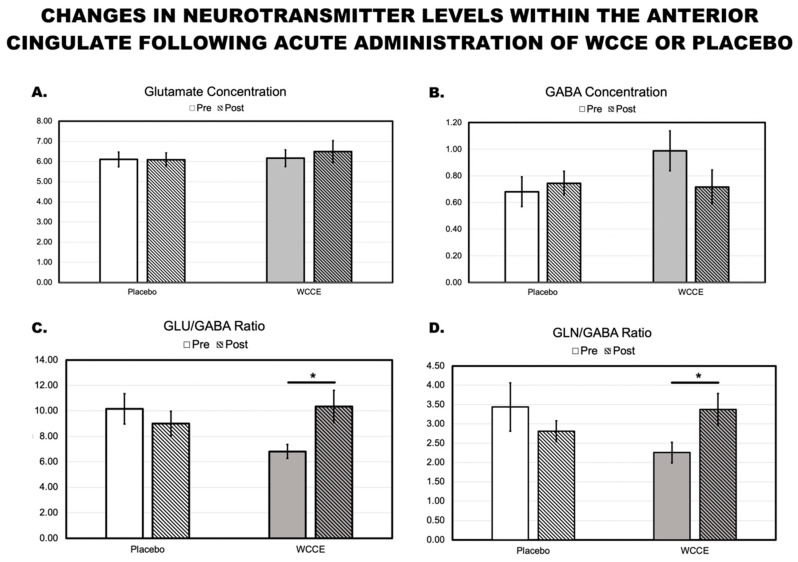
Bar graph with M ± SEM demonstrating the changes in glutamate, gamma-aminobutyric acid (GABA), and the ratios of glutamate/GABA (GLU/GABA) and glutamine/GABA (GLN/GABA) within the anterior cingulate cortex following the administration of either the placebo or WCCE. Y-axis units of measurement are institutional units. A * indicates significance at the *p* < 0.05 level for one-tailed *t*-tests. For descriptive data, please see the supplemental table hosted at https://osf.io/qypr8/. Clear bars represent the placebo, while gray bars represent WCCE. Striped bars indicate post-measurements.

Treatment differences were not observed for GABA or glutamate independently. Notably, WCCE showed a qualitative decrease in GABA that approached significance. However, both the glutamate/GABA ratio (WCCE: *t*(6) = 2.192; *p* = 0.036; estimated Cohen’s *d* = 0.83, one-tailed; placebo: *t*(6) = −0.978; *p* > 0.05) and the glutamine/GABA ratio (WCCE: *t*(6) = 2.155; *p* = 0.038, estimated Cohen’s *d* = 0.82, one-tailed; placebo: *t*(6) = −1.323; *p* > 0.05) were significantly different post-consumption of WCCE but not the placebo. Specifically, WCCE demonstrated a significant increase in glutamate/GABA (pre-WCCE (M ± SEM): 6.81 ± 0.54; post-WCCE: 10.35 ± 1.26) and glutamine/GABA ratios (pre-WCCE: 2.26 ± 0.27; post-WCCE: 3.37 ± 0.41), while the placebo demonstrated no change (glutamate/GABA pre-placebo: 10.16 ± 1.20; post-placebo: 9.00 ± 0.97; glutamine/GABA pre-placebo: 3.44 ± 0.63; post-placebo: 2.81 ± 0.71) and qualitatively decreased post-consumption (Figure 6). All descriptive data are presented in the supplemental tables hosted at https://osf.io/qypr8/.

### 3.4. BDNF

**Figure 7:**
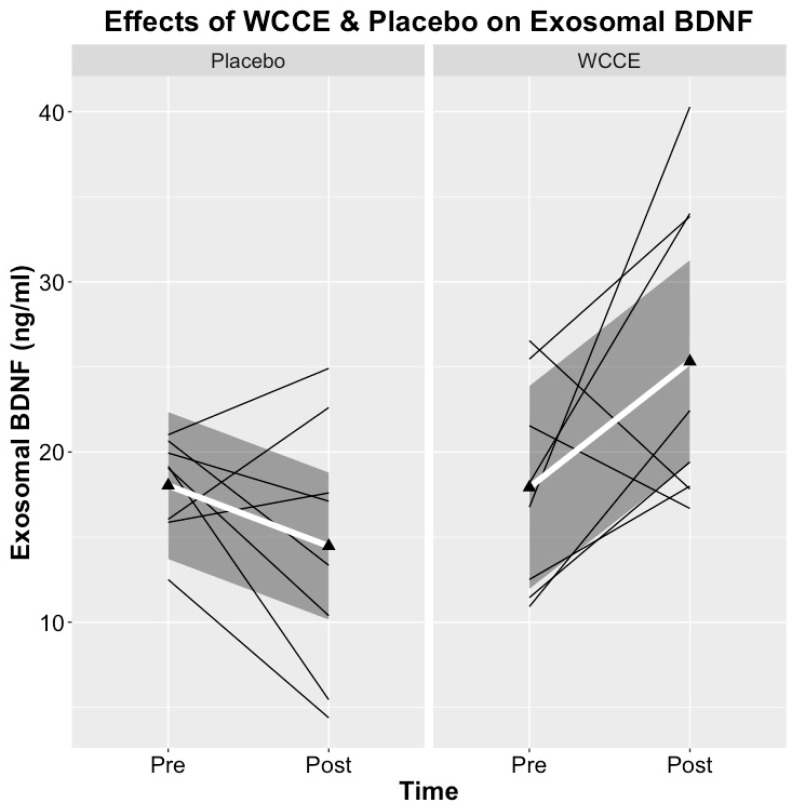
Pre- and post-consumption data for exosomal brain-derived neurotrophic factor (BDNF). Thin black lines indicate individual participant data, while the thicker white line depicts the group average. The dark gray shading surrounding the white line uses locally weighted regression (loess) for a visualization of group variability. Line graphs were created with open-source software ggplot2 in R.

Despite no differences in exosomal BDNF at baseline between treatment days, there was a significant increase in exosomal BDNF after the ingestion of WCCE when compared to pre-WCCE ingestion (*t*(7) = 2.00; *p* = 0.04, one-tailed; estimated Cohen’s *d* = 0.71 (Figure 7, Table 2). No differences in exosomal BDNF were observed post-placebo ingestion when compared to pre-placebo baseline levels (*t*(7) = 1.855; *p* > 0.05, one-tailed).

### 3.5. Behavioral Results

For both the go/no-go and n-back tasks, we examined reaction time and accuracy. For reaction time, we found significant reductions on correct trials during the n-back task post-consumption for WCCE (*t*(7) = −3.649; *p* = 0.004; estimated Cohen’s *d* = −1.29, one-tailed) but not for the placebo (*t*(7) = −0.406; *p* > 0.05). No significant differences were identified for reaction time for the go/no-go task. For accuracy, we found that accuracy during the go/no-go task declined post-consumption of the placebo (*t*(7) = −2.758; *p* = 0.014; estimated Cohen’s *d* = 0.98, one-tailed) but not post-consumption of WCCE (*t*(7) = −0.406; *p* > 0.05) (Table 3), thus suggesting a possible protective effect of WCCE. No significant differences were noted during the n-back task with regard to accuracy.

### 3.6. HPLC Analyses

An analysis of the antioxidant capacity of the 5 assays (ORAC, HORAC, NORAC, SORAC, and SOAC), as well as the total value, is presented in Table 4.

## 4. Discussion

Here, we used advanced neuroimaging techniques to identify neurophysiological changes associated with a polyphenol-rich and unique material, WCCE. Following the ingestion of a single dose, our data demonstrated neurophysiological changes concomitant with behavioral improvements. To our knowledge, this represents the first comprehensive study of its kind assessing multi-level outcomes employing a rigorous double-blind, randomized, within-subjects crossover design.

Before discussing the outcome of our acute administration clinical trial, it is important to note the chemical composition of WCCE and its associated antioxidant properties. For this, we report the outcome of a series of five antioxidant assays to characterize the antioxidant properties of WCCE. Using LC–MS, a recent study demonstrated that WCCE is rich in chlorogenic acid compounds and other polyphenols [27], which was in line with previous research by Mullen and colleagues (2011) [30], who had identified 24 such compounds including hydroxycinnamate, flavan-3-ols, and flavonol conjugates in samples of arabica and robusta coffee extracts. Here, we demonstrate that the robust polyphenol profile of WCCE is concomitant with notable antioxidant properties that are consistent with hypothesized mechanisms underlying the neurotrophic and potentially therapeutic effects of polyphenols [21,25,26]. This is particularly important because of the causal links between oxidative stress and inflammation in neurodegeneration and brain aging [5,26,82].

Our primary objective was to examine changes in neurophysiological markers as a result of acute WCCE administration. As a major neurotrophic factor that serves several important regulatory roles in the nervous system, including neuronal development, synaptic plasticity, and neuronal survival, BDNF was postulated to increase following the administration of WCCE. Several studies have shown that exosomes carry neuronal proteins which are able to cross the blood–brain barrier. However, other than one previous report, little is known concerning the presence of BDNF in exosomes in response to nutritional supplementation. Here, we found that exosomal levels of BDNF were increased by roughly 41% in exosomes obtained from the serum of study participants after single-dose WCCE ingestion, whereas the placebo resulted in no significant change. Importantly, polyphenols have had a consistent, robust effect on BDNF in both human and animal models [9,23,25,26].

One mechanism through which BDNF appears to maintain elevated levels of neuronal excitation is through the prevention of GABAergic signaling activities [83]. While glutamate is the brain’s major excitatory neurotransmitter and phosphorylation normally activates receptors, GABA is the brain’s primary inhibitory neurotransmitter. The phosphorylation of GABA_A_ receptors tends to reduce their activity. Therefore, increases in exosomal BDNF after the ingestion of WCCE may suggest a relationship with the trends observed in the present study for a decrease in GABA and an increase in glutamate (Figure 5). This may also reflect the glutamate/GABA balance, which has been shown in previous studies to be associated with BDNF [36]. Indeed, we did find significant differences in the glutamate/GABA ratio and the glutamine/GABA ratio. Specifically, WCCE led to greater increases in the ratio post-consumption, suggesting that either glutamine increased, GABA decreased, or a combination of the two occurred. In contrast, the placebo caused no significant change, with qualitative decreases noted. This is an intriguing result, especially given that glutamine has been shown to be a useful predictor of prognosis in psychiatric conditions and has been associated with a number of roles including as a precursor for neurotransmitter and protein synthesis [84,85,86,87,88]. Together with our other observations, these data suggest the bioavailability and bioactivity of the (as yet not fully identified) active principals within WCCE. Additional studies will need to be conducted to further explore these relationships.

While glutamate and GABA have been implicated in BDNF levels, we failed to find significant effects when testing the individual neurotransmitter changes. We did observe trends wherein WCCE increased glutamate and decreased GABA, and we found a significant change in the ratios between these two neurotransmitters. This latter result suggests that WCCE likely has an effect on the glutamine–glutamate cycling. Given that aging has been associated with aberrations in glutamate, GABA, and glutamate/GABA ratios, these results are clinically intriguing [89,90] and should be corroborated by larger studies. It is of note, however, that placebo pre-baseline values were similar to post-administration WCCE values. As such, it is possible that post-WCCE administration was a function of regression to the mean baseline. While we do not suspect this to be the case given the within-subject nature of the design, it is still worthy of consideration and notable for future studies. Furthermore, in exploratory analyses with other neurometabolites/neurotransmitters (data not shown here), we noted differences in taurine that approached significance as a result of WCCE ingestion (M ± SEM: 1.26 ± 0.05 pre-ingestion versus 1.43 ± 0.09 post-ingestion; *t*(7) = 1.881; *p* = 0.10, two-tailed). Taurine affects neuronal signaling through its interactions on ion channels, particularly calcium, and has been demonstrated to protect against inflammation, apoptosis, and oxidative stress in animals [91,92], all of which have been associated with polyphenols. Additionally, taurine has known effects on GABA receptors, potentially providing a mechanistic explanation for the effects on BDNF. While these inferences should be interpreted with caution, future studies should examine the effects of WCCE and other polyphenol-rich materials on taurine and other neurometabolites.

We also used submillimeter fMRI to identify neurofunctional changes associated with WCCE ingestion. During most clinical investigations, there is an expectation that any tested substance will increase the activity of some physiological or psychological parameter and that the placebo will do little or nothing. Consequently, we found it to be interesting to observe that, under these experimental conditions, our data suggested that WCCE actually reduced BOLD activation throughout fronto-limbic regions, while, in contrast, the placebo increased activity in key structures involved in decision making, suggesting that WCCE may increase the efficiency of pivotal regions contributing to cognition. Specifically, across conditions of the go/no-go task, post-WCCE administration led to less activation in the left inferior frontal gyrus and left insula. In contrast, post-placebo administration was associated with an increased activity in the cingulate and posterior cingulate cortex. These stark post-consumption differences represent an initial finding that WCCE may have distinct neurophysiological effects that should be corroborated with larger studies. Additionally, it should be noted that we did observe increases in activation following the consumption of WCCE within the right posterior cingulate and right parahippocampal gyrus during the rest periods of the n-back task, while we did not find any differences during the task. Furthermore, during the n-back task, we observed connectivity differences such that WCCE was associated with increased connectivity between the ACC and regions involved in working memory, error monitoring, and sensory processing. The latter is particularly interesting given recent results demonstrating changes in measures of concentration and fatigue [10], as well as cognitive improvements in an n-back paradigm noted in a longitudinal study [35]. During the same task, the placebo was associated with greater functional connectivity between the ACC and the precuneus, a region linked to very discrete sensory processing. Coupled with our behavioral results, WCCE appears to affect cognitive processing, including working memory and response inhibition, through specific neurofunctional changes within fronto-limbic and fronto-parietal networks and the altered recruitment of these networks through connectivity changes with neural hubs associated with these networks. Further studies with larger samples will be needed to confirm these findings.

Finally, we examined behavioral performance on two well-known tasks of cognition—the n-back and the go/no-go task. Behaviorally, WCCE resulted in an approximately four times greater reduction in reaction time during the n-back task compared to the placebo. Furthermore, the placebo was associated with a decreased accuracy during the go/no-go task, with participants committing over 50% more errors compared to post-WCCE ingestion. This manifestation within the placebo group may have been due to mental fatigue, decreased alertness, or increased frustration in individuals who, by definition, were untreated and were not availed of the support of the WCCE. These results suggest that WCCE has acute behavioral effects and further corroborates findings demonstrating long-term effects of WCCE administration [35]. Moreover, these acute results may imply longer-term significant effects on cognitive processing, pointing toward an increased efficiency (i.e., reduced reaction time) and potentially protecting against errors through increased sustained attention or other mechanisms. This has been behaviorally supported in recent research [35], also conducted in older adults.

Taken together, our results contribute additional evidence linking polyphenols with significant antioxidant properties to positive neurophysiological and behavioral outcomes in an aging population with subjective cognitive decline. Our data represent the first of their kind examining a nutraceutical using a multimodal approach. Additional studies will be necessary to further characterize the effects of polyphenol-rich compounds on the brain.

### Limitations

The current study had several notable limitations. First, the sample size was small (*n* = 8), and both men and women participated. While we leveraged a within-subjects design, future studies should seek larger samples and should explore sex differences. However, it should also be noted that recent studies have highlighted the utility of smaller samples with more robust measurement designs. Specifically, Laumann and colleagues (2015) [45] pointed out that large, group-based functional neuroimaging analyses may hide significant individual variability that could be particularly meaningful. Similar results have been noted in a variety of studies [42,43,44,45], suggesting that controlling for individual variability by way of within-subjects designs may substantially improve power, especially in consideration of robust, multi-modal functional neuroimaging studies. Second, because we used a truly random assignment procedure, we had an unbalanced ordering (six received WCCE first and only two received the placebo first). However, one would expect that decreases in reaction time and improvements in accuracy would occur with practice; thus it could be argued that placebo conditions would be at an advantage given that participants would have been overly familiar with the tasks at the time of their fourth administration (i.e., post-placebo ingestion). Thus, we feel confident that the observed behavioral effects were robust, especially since they corroborated with previous evidence [10,35]. Nonetheless, it will be important to conduct larger samples with balanced ordering so that more strict statistical models can be employed (e.g., multivariate analysis of covariance (MANCOVA)). Third, this study was limited to the assessment of observed activities within 90 min following a single dose of WCCE/placebo. Longer term studies may yield a deeper understanding of potential functional benefits and the underlying mechanisms involved. Fourth, we did not control the participants’ diets, nor did we evaluate their habits. However, we have no reason to believe that their diets or habits changed between sessions. Regardless, additional studies should employ diaries or other accounts to examine these important lifestyle factors. Finally, it should be noted that numerous bioactive compounds within WCCE could be causing the results demonstrated in this study. Investigations to determine the active principles within WCCE could provide further insight into mechanisms of action and could lead to a greater specificity and amplitude of responses. This could include animal studies that would allow for the more precise identification of potential mechanisms of action. Future studies should employ parametric designs to more specifically assess the effects of unique polyphenol-rich materials on cognition and neurophysiology.

## 5. Conclusions

To the best of our knowledge, this is the first time that, aside from traditional psychological assessments such as n-back or go/no-go, any well-characterized and quantified dietary supplement material has been reported to induce measurable, acute physiological changes in brain connectivity, as well as potential changes in certain neurometabolites. Here, we demonstrated significant improvements in cognition, with concomitant observed changes in neurofunctional brain networks after a single 100 mg dose of WCCE, a polyphenol-rich plant-based extract that appears to increase exosomal BDNF, an essential neuroprotein. Finally, using a multi-modal approach, we present a robust and potentially useful methodology for the bigger-picture evaluation of candidate materials with possible application for the modulation of neuropsychophysiological activity. Additional well-powered studies should be conducted for a full assessment of WCCE’s effects and mechanisms of action.

## Figures and Tables

**Table 1:** List of polyphenol compounds in WCCE, expressed in mg/g ± SD. Table adapted from the work of Nemzer et al. (under review) [27].

Polyphenol	mg/g
3-*O*-Caffeoylquinic acid	41.3 ± 8.4
5-*p*-coumaroylquinic acid	0.4 ± 0.1
5-*O*-Caffeoylquinic acid	134.6 ± 16.7
3-Feruloylquinic acid	7 ± 1.2
4-*O*-Caffeoylquinic acid	74.9 ± 11.1
*p*-coumaroylquinic acid	0.6 ± 0.1
*p*-coumaroylquinic acid	2.5 ± 0.5
4-Feruloylquinic acid	8.4 ± 1.7
5-Feruloylquinic acid	38.1 ± 6.3
3-*O*-Caffeoylquinic lactone	7.8 ± 1.2
4-*O*-Caffeoylquinic lactone	3.8 ± 0.6
3,4-*O*-Dicaffeoylquinic acid	37.6 ± 7.3
3,5-*O*-Dicaffeoylquinic acid	12.1 ± 2.2
4,5-*O*-Dicaffeoylquinic acid	47.2 ± 7.8
3-*O*-Feruloyl-4-Caffeoylquinic acid	0.9 ± 0.2
3-*O*-Caffeoy-4-Feruloylquinic acid	3.3 ± 0.6
3-*O*-Feruloyl-5-Caffeoylquinic acid	0.2 ± 0
3-*O*-Caffeoy-5-Feruloylquinic acid	1.1 ± 0.3
4-*O*-Feruloyl-5-Caffeoylquinic acid	0.7 ± 0.2
4-*O*-Caffeoy-5-Feruloylquinic acid	3.4 ± 0.7
Total CGA	425.8 ± 63.9
Trigonelline	33.78 ± 5.2
Caffeine	18.2 ± 3.3

**Table 2:** Descriptive statistics of exosomal BDNF. Results indicated a significant increase in the exosomal BDNF level after the ingestion of WCCE when compared to pre-WCCE ingestion. No differences in exosomal BDNF were observed post-placebo ingestion when compared to pre-placebo baseline levels. M = mean, SD = standard deviation, SEM = standard error of the mean.

Exosomal BDNF Results														
	Descriptives						t-Statistics							
	Pre			Post						95% Confidence Interval of the Difference				
	M	SD	SEM	M	SD	SEM	M	SD	SEM	Lower	Upper	t	df	Sig. (1-Tailed)
Post WCCE> Pre WCCE	17.92	6.17	2.18	25.31	9.26	3.27	7.40	10.45	3.69	−1.34	16.13	2.00	7	0.04
Post Placebo > Pre Placebo	18.03	2.95	1.04	14.47	7.50	2.65	−3.56	7.08	2.50	−9.48	2.36	−1.42	7	0.10

**Table 3:** Descriptive statistics of the behavioral tasks (i.e., n-back and go/no-go) performed in the scanner. Notably, for reaction time, we found significant reductions during the n-back task post-consumption for WCCE but not for the placebo. For accuracy, we found that accuracy during the go/no-go task declined post-consumption of the placebo but not post-consumption of WCCE.

**N-back Behavioral Results**															
		**Descriptives**						**t-Statistics**							
		**Pre**			**Post**						**95% Confidence Interval of the Difference**				
		**M**	**SD**	**SEM**	**M**	**SD**	**SEM**	**M**	**SD**	**SEM**	**Lower**	**Upper**	**t**	**df**	**Sig. (1-Tailed)**
Post WCCE > Pre WCCE	Number of Errors	2.125	2.532	0.895	2.625	2.774	0.981	0.500	2.070	0.732	−1.231	2.231	0.683	7	0.258
	Reaction Time for Correct Trials	574.670	84.335	29.817	536.527	82.185	29.057	−38.144	29.567	10.454	−62.863	−13.425	−3.649	7	0.004
Post Placebo > Pre Placebo	Number of Errors	3.250	3.770	1.333	3.000	2.828	1.000	−0.250	1.389	0.491	−1.411	0.911	−0.509	7	0.313
	Reaction Time for Correct Trials	568.838	111.814	39.532	559.269	86.193	30.474	−9.569	66.678	23.574	−65.314	46.175	−0.406	7	0.349
**Go/No-Go Behavioral Results**															
		**Descriptives**						**t-Statistics**							
		**Pre**			**Post**						**95% Confidence Interval of the Difference**				
		**M**	**SD**	**SEM**	**M**	**SD**	**SEM**	**M**	**SD**	**SEM**	**Lower**	**Upper**	**t**	**df**	**Sig. (1-Tailed)**
Post WCCE > Pre WCCE	Reaction Time for Successful Trials	387.953	38.729	15.811	381.654	44.676	18.239	−6.299	23.166	9.457	−30.610	18.011	−0.666	5	0.2675
	Accuracy	242.000	5.967	2.436	242.333	4.082	1.667	0.333	2.805	1.145	−2.610	3.277	0.291	5	0.3915
Post Placebo > Pre Placebo	Reaction Time for Successful Trials	397.013	26.611	9.408	395.563	41.291	14.599	−1.450	25.287	8.940	−22.591	19.691	−0.162	7	0.438
	Accuracy	242.125	5.463	1.931	239.625	7.170	2.535	−2.500	2.563	0.906	−4.643	−0.357	−2.758	7	0.014

**Table 4:** Antioxidant analysis of WCCE. Results are expressed as µmol Trolox equivalent/g ± SD. ORAC: oxygen radical absorbance capacity.

Antioxidant Assay	µmol Trolox Equivalent/g
ORAC	6097 ± 225
HORAC	18,709 ± 426
NORAC	527 ± 52
SORAC	860 ± 24
SOAC	2042 ± 185
Total ORAC	28,237 ± 782

## Data Availability

The data presented in this study are openly available at https://osf.io/qypr8/.

## Supplementary Materials

The following are available online at https://osf.io/qypr8/, Table S1: Differences in the Go/No-Go Task (All Trials Combined), Table S2: Differences in Go Trials During the Go/No-Go Task, Table S3: Differences in No-Go Trials During the Go/No-Go Task, Table S4: Differences in No-Go Trials During the Go/No-Go Task, Table S5: No-Go > Go Trial Differences, Table S6: Differences in Rest During the N-back Task, Table S7: Neurometabolite (MRS) Results.
